# The heat shock response restricts virus infection in *Drosophila*

**DOI:** 10.1038/srep12758

**Published:** 2015-08-03

**Authors:** Sarah H. Merkling, Gijs J. Overheul, Joël T. van Mierlo, Daan Arends, Christian Gilissen, Ronald P. van Rij

**Affiliations:** 1 Department of Medical Microbiology, Radboud Institute for Molecular Life Sciences, Radboud University Medical Center, PO Box 9101, 6500 HB Nijmegen, The Netherlands; 2 Department of Human Genetics, Radboud University Medical Center, Nijmegen, The Netherlands

## Abstract

Innate immunity is the first line of defence against pathogens and is essential for survival of the infected host. The fruit fly *Drosophila melanogaster* is an emerging model to study viral pathogenesis, yet antiviral defence responses remain poorly understood. Here, we describe the heat shock response, a cellular mechanism that prevents proteotoxicity, as a component of the antiviral immune response in *Drosophila*. Transcriptome analyses of *Drosophila* S2 cells and adult flies revealed strong induction of the heat shock response upon RNA virus infection. Dynamic induction patterns of heat shock pathway components were characterized *in vitro* and *in vivo* following infection with different classes of viruses. The heat shock transcription factor (*Hsf*), as well as active viral replication, were necessary for the induction of the response. *Hsf*-deficient adult flies were hypersensitive to virus infection, indicating a role of the heat shock response in antiviral defence. In accordance, transgenic activation of the heat shock response prolonged survival time after infection and enabled long-term control of virus replication to undetectable levels. Together, our results establish the heat shock response as an important constituent of innate antiviral immunity in *Drosophila*.

Innate immunity is a complex, heritable line of defence shared by all metazoans^1^. Innate immune pathways of the fruit fly *Drosophila melanogaster* are evolutionary conserved with those of mammals^1^, and the wide range of genetic tools available in this system have been instrumental in the discovery of fundamental aspects of antimicrobial immunity^2^. Over the past decade, the fly model has also been used for the discovery and characterization of antiviral immune pathways^3^,^4^, yet, the host response to virus infection in insects remains incompletely understood.

In mammals, sensing of viral infections is mediated by pattern recognition receptors that recognize virus-associated molecules. For example, viral double-stranded RNA is recognized by Toll- or RIG-I-like receptors, leading to the activation of the NF-κB and IRF3/IRF7 transcription factors that induce the expression of antiviral type I interferons and other proinflammatory cytokines^5^,^6^. Type I interferons activate the Jak-Stat pathway to induce expression of a wide array of antiviral effectors in infected and non-infected cells^5^,^6^. Also in *Drosophila*, antiviral immunity is mediated by the Jak-Stat pathway, through the activation of general stress-induced genes, as well as the virus-specific gene *vir-1*, whose function remains to be determined^7^,^8^.

Other evolutionary conserved antiviral mechanisms have recently been described. Autophagy, a highly conserved catabolic pathway that responds to nutrient starvation, mediates immunity against a range of viruses in flies and mammals^9^,^10^. RNA interference (RNAi) is a broadly active antiviral mechanism in insects. Sensing of viral double-stranded RNA by Dicer-2 precedes its cleavage into small interfering RNAs (siRNAs), which, in association with the Argonaute-2 containing RNA induced silencing complex (RISC), mediate degradation of complementary viral RNA molecules^4^,^11^. In mammals, RNAi has been reported to have antiviral activity in specific cell lineages^12^,^13^.

The heat shock response is a cellular pathway that is activated by a myriad of stressors, including extreme temperature, chemicals, and physical injuries, which are all associated with the accumulation of damaged proteins. These stimuli lead to the activation of heat shock transcription factors (Hsf) in a multi-step process that includes trimerization and translocation of Hsf to chromosomal target loci, among which are promoters of genes encoding heat shock proteins (Hsp)^14^. The *Drosophila* genome encodes a single Hsf, which relocates upon activation from the nucleoplasm to regulatory regions of Hsp genes and triggers their rapid transcription^15^,^16^. Heat shock proteins are molecular chaperones that mediate protein folding and re-folding, modulate entry into proteasomal and autophagic degradation pathways, or interact with metabolic stress sensors, thereby preventing global cellular stress and proteotoxicity^17^,^18^. There are several families of heat shock proteins, classified by their molecular mass and sequence conservation. The main constituents are Hsp90/Hsp100 (called Hsp83 in *Drosophila*), Hsp70, Hsp40, and the small Hsps. Small heat shock proteins, such as Hsp23, Hsp26 and Hsp27 in *Drosophila*, range in size from 10 to 40 kDa, and have, in contrast to the ubiquitous Hsp70 and Hsp90, distinct developmental, tissue, and subcellular expression patterns^17^.

In addition to abiotic stresses, microbial infections may also induce the heat shock response^19^. In mammals, fever activates the heat shock response, which then functions through several mechanisms, including direct inhibition of pathogen growth or cytoprotection of host cells. Fever may also induce bacterium-encoded Hsps and both host and pathogen-derived Hsps can activate the immune response of the host^20^,^21^. However, the heat shock response can also be exploited by pathogens for their own replication^22^. For instance, Hsp70 participates in entry of rotaviruses into host cells^23^, uncoating of adenoviruses in the cytoplasm^24^, and folding and maturation of picornavirus capsid proteins^25^. Whether the benefit of heat shock responses can be attributed to either the host or the pathogen seems to depend on the nature and context of their interaction^22^. As most of these observations originate from studies in cell culture systems, the importance of the heat shock response *in vivo* remains to be elucidated.

In this study, we investigated the role of the cellular heat shock response upon viral infection in *Drosophila*. Analyses of *in vitro* and *in vivo* transcriptome data revealed strong induction of the heat shock response upon viral challenge. *Hsf*-deficient flies that lack the heat shock response were hypersensitive to virus infection. Conversely, transgenic activation of the response resulted in prolonged control of virus replication and reduced virus-induced mortality. Taken together, our results indicate that the heat shock response is an important component of antiviral defence in flies.

## Materials and Methods

### Fly strains and husbandry

Flies were raised on standard cornmeal-agar medium at 25°C in a light/dark cycle of 12 h/12 h. The *Hsf *^4^ mutant was obtained from the Drosophila Genetic Resource Center (stock no. 108–256) and the *Hsf *^4^*; Hsf*^+^ rescue line from the Bloomington Stock Center (stock no. 5490, described in ref. 26). *CnBw*, which is the genetic background for those lines, was used as the wild-type control in all experiments. The following fly lines have been described previously: *Hml-Gal4* (ref. 27), *C564-Gal4* (ref. 28), *UAS-Hsp70* (ref. 29), *UAS-Hsf* (ref. 16). The *Hsf*^RNAi^ line expressing a short hairpin targeting *Hsf*, and the driver *Act-Gal4* were obtained from the Bloomington Stock Center (stock no. 27070 and 4414, respectively). The genetic background of wild-type flies used for RNA sequencing is *y*^1^*w*^1^, and has been described previously as *EHMT*^+^ (ref. 30) or *G9a*^+/+^ (ref. 31). *In vivo* RNAi experiments were performed by crossing GMR-Gal4, UAS-*th*^RNAi^/CyO virgins^32^ with *Hsf*^RNAi^ male flies or with control flies containing the attP landing site used to introduce the RNAi-inducing transgene *(y*^1^*v*^1^*; attP2*; Bloomington stock no. 36303). The eye phenotype was assessed in three to five-day-old female F1 offspring lacking the CyO balancer.

### Virus infection

*Drosophila* S2 cells (Invitrogen) were maintained in Schneider’s *Drosophila* medium (Gibco) containing 10% heat-inactivated Fetal Bovine Serum (Gibco), 50 U/mL Penicillin and 50 μg/mL Streptomycin (Gibco). Cells were seeded in 6-well plates at a density of 2.5 × 10^6^ cells/mL in 2 ml medium, and infected with Drosophila C Virus (DCV), Cricket paralysis virus (CrPV), or Invertebrate iridescent virus (IIV-6) at an MOI (Multiplicity of Infection) of 10. After removal of the medium, S2 cells were incubated with virus inoculum in 0.5 mL of fresh Schneider’s medium for an hour, and the inoculum was subsequently replaced with 2 mL of fresh medium. Cells were harvested in 1 mL of Isol-RNA Lysis Reagent at given time points. UV-inactivated virus stocks were generated by exposing the virus inoculum to a total of 24,000 mJ of UV light in eight intervals of 90 sec in a GS Gene linker UV Chamber (Bio-Rad).

Fly stocks were cleared of *Wolbachia* and persistent virus infections as described previously^33^,^34^,^35^. Three to five-day-old flies were injected intrathoraxically with a Nanoject II injector (Drummond) after anesthesia with CO_2_. Injection doses were 1,000 median tissue culture infectious dose (TCID_50_) units of DCV and CrPV, and 14,000 TCID_50_ units of IIV-6 in 10 mM Tris-HCl, pH 7.3. Survival was assessed daily and flies were transferred to fresh food every 3 days. Lethality at day 1 was attributed to the injection procedure and excluded from the survival analysis. Unless noted otherwise, three pools of 10 to 15 flies were injected per condition with independent dilutions of virus stock.

### Virus titration

Viral titers were determined by end-point dilution, as described previously^33^. In short, ten-fold dilutions of fly homogenates were used to inoculate 2 × 10^4^ S2 cells (Invitrogen) per well in 96-well plates in quadruplicate. After 5 days, cells were transferred to fresh medium, and cytopathic effect (CPE) was monitored until day 14. Viral titers were calculated using the method of Reed and Muench^36^.

### *In vivo* RNAi reporter assay

RNAi competency of adult flies was analysed using a reporter assay, as described previously^34^. Briefly, three to five-day-old female flies were injected in the abdomen with a 100 nl suspension containing a 1:1 mixture of Schneider’s *Drosophila* Medium (Gibco) and Lipofectamine 2000 (Invitrogen) complexed with Firefly luciferase (Fluc) and Renilla luciferase (Ren) reporter plasmids, and Fluc specific or non-specific control dsRNA (GFP). After incubation for 3 days, Fluc and Ren activity was measured in fly homogenate using the Dual Luciferase assay reporter system (Promega). Fluc over Ren ratios were calculated for each sample, and data are presented as fold silencing relative to the non-specific dsRNA control.

### RNA analysis

RNA was isolated from S2 cells or flies using Isol-RNA lysis Agent (5-Prime). cDNA synthesis was performed on 1 μg RNA, pre-treated with DNase I (Ambion), using TaqMan Reverse Transcription reagents (Applied Biosystems) according to the manufacturer’s instructions. qPCR was performed with SYBR Green I Master Mix on a LightCycler 480 (Roche). The qPCR program was the following: 95 °C for 5 min, followed by 45 cycles of 95 °C for 5 s, 60 °C for 10 s, 72 °C for 20 s. Expression of the gene of interest was normalized to transcript levels of the housekeeping gene *Ribosomal Protein 49* (Rp49), and fold change relative to mock infection was calculated using the ΔΔCt method^37^. Rp49 Ct values were stable in all conditions and experiments. The primers used for qPCR analysis are provided in Supplementary table S1.

### Microarray

S2 cells were infected with DCV (MOI = 10) or mock infected (Schneider’s medium), and 3 biological replicates were collected at 8 and 24 hours post-infection (hpi). RNA was extracted with the RNeasy Mini Kit (Qiagen) followed by on-column DNaseI treatment, according to the manufacturer’s instructions. RNA was labelled using the GeneChip WT Terminal Labelling kit (Affymetrix), and hybridized on an Affymetrix Drosophila GeneChip microarray 2.0 for 17 hours at 45 °C in the Affymetrix hybridization oven at 60 rpm. The arrays were washed and stained on a Fluidics station 450 according to Affymetrix protocol FS450_0001 and analysed on a GeneChip scanner 3000 7G. Partek software was used for RMA background correction and statistical analyses. An Analysis of Variance (ANOVA) was performed on the three conditions followed by a post-hoc analysis comparing the individual conditions to each other. *P*-values were corrected for multiple testing by a step-up False Discovery Rate (FDR). Gene induction was calculated relative to a mock infection at 8 hpi. The dataset is available at the NCBI Gene Expression Omnibus under series accession number GSE57434.

### RNA sequencing

Thirty female flies (3–5 days old) were collected at 24 hpi with 10,000 TCID_50_ units of DCV or CrPV, and RNA was isolated using Isol-RNA Lysis reagent as described above. Procedures for library preparation, sequencing, and data analyses have been published^34^. The RNA-Seq data are available at the NCBI Gene Expression Omnibus under accession number GSE56013.

### Western Blotting

S2 cells in 6-well plates were infected with DCV at an MOI of 10 as described above. Cells were harvested at different time points by resuspension in PBS (Phosphate Buffer Saline) buffer and centrifugation for 5 minutes at 2,000 × g. Supernatant was discarded and the cell pellet was resuspended in Laemmli sample buffer (4% SDS, 20% glycerol, 120 mM Tris-HCl pH = 6.8, 0.02% bromophenol blue). The samples were separated on a 12.5% SDS-PAGE gel and transferred to a nitrocellulose membrane (pore size 0.2 μm, Bio-Rad). The membrane was incubated with rat anti-Hsp70 antibody (SMC-230D, StressMarq), followed by an incubation with IRDye 680-conjugated goat anti-rat IgG antibody (LI-COR). The proteins were visualized with an Odyssey infrared imager (LI-COR).

### Statistical analysis

Unpaired two-tailed Student’s t-tests and Wilcoxon rank-sum tests, as implemented in Graphpad Prism version 6, were used to compare differences in gene expression and log-transformed viral titers and single-fly viral RNA levels, respectively. Survival assays were assessed using Kaplan-Meier analyses and log-rank tests, as implemented in SPSS Statistics (version 20, IBM). *P*-values below 0.05 were considered statistically significant. Enrichment of Gene Ontology terms was analysed in GoToolBox^38^, using the hypergeometric test with Benjamini & Hochberg correction.

## Results

### The heat shock response is induced in DCV-infected *Drosophila* S2 cells

**Figure 1:**
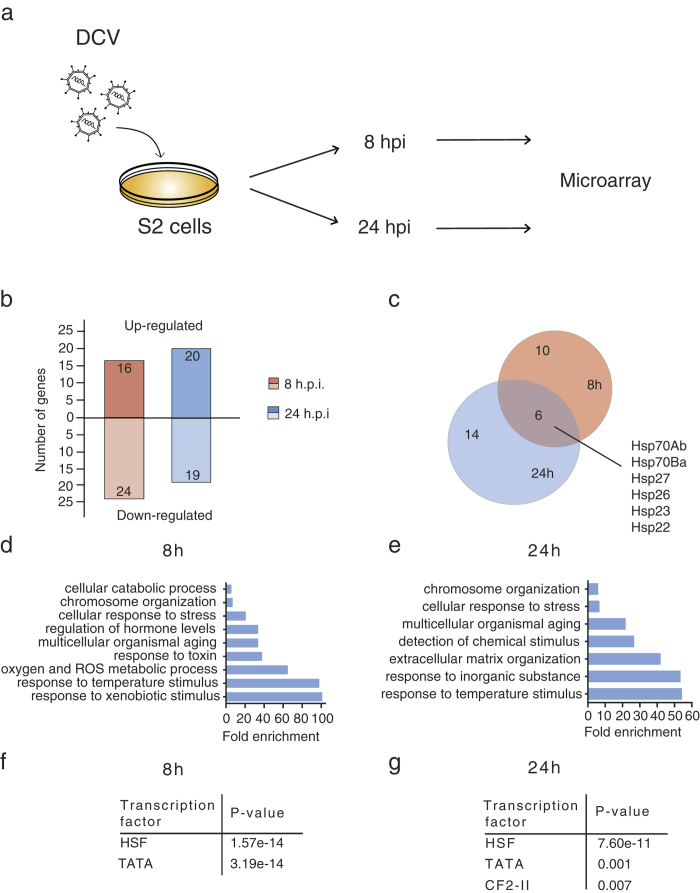
Microarray analysis of DCV-infected *Drosophila* S2 cells. (**a**) Overview of the experimental workflow. S2 cells were infected with DCV (MOI = 10) or mock-infected with Schneider’s medium, and RNA was extracted at 8 and 24 hours post-infection (hpi) for microarray analyses. Figure drawn by S.H. Merkling. (**b**) Number of differentially expressed genes at 8 and 24 hpi (fold change ≥2 relative to mock infection). (**c**) Venn diagram representing the overlap between differentially induced genes after DCV infection at 8 and 24 hpi. (**d**,**e**) Gene ontology (GO) analysis of the genes that are upregulated ≥2-fold at (**d**) 8 hpi and (**e**) 24 hpi. All significantly enriched level 4 GO terms are shown (*P* < 0.05 in a hypergeometric test with Benjamini & Hochberg correction). (**f**,**g**) Enrichment of predicted transcription factor binding sites amongst genes induced ≥2-fold at (**f**) 8 hpi and (**g**) 24 hpi. The 500-bp region upstream of the transcriptional start site was analysed in Pscan, using the Transfac database. All transcription factors that are significantly enriched over the genome-wide mean are shown (*P* < 0.05 in a z-test).

To identify novel factors or processes involved in antiviral defence in *Drosophila*, we generated transcriptional profiles of DCV-infected *Drosophila* S2 cells at 8 and 24 hours post-infection (hpi) using Affymetrix GeneChip microarrays (Fig. 1a). S2 cells are likely derived from hematopoietic tissues and have macrophage-like properties; they can therefore be described as hemocyte-like^39^. Even though S2 cells support efficient DCV replication, we noted that only a limited number of genes were significantly induced by DCV infection relative to mock (n = 16 and n = 20 at 8 and 24 hpi, respectively, Fig. 1b, Supplementary Figure S1). Overlap of the upregulated genes at both time points revealed that 6 genes, all encoding members of the heat shock protein family (*Hsp70Ab*, *Hsp70Ba*, *Hsp22*, *Hsp23*, *Hsp26*, *Hsp27*), were consistently induced upon infection (Fig. 1c). Accordingly, Gene Ontology (GO) term analysis showed that terms such as “chromosome organization”, “response to temperature stimulus” or “cellular response to stress” were significantly enriched at 8 and 24 hpi (Fig. 1d,e). Likewise, prediction of transcription factor binding sites in the promoter regions of the upregulated genes detected strong enrichment of heat shock factor (Hsf) binding motifs at both time points (Fig. 1f,g). In addition, the TATA box and the binding motif for Chorion factor 2 were enriched at both time points, suggesting that genes under control of these elements are also activated upon viral challenge. Additionally, we observed that relatively few genes were downregulated at 8 and 24 hpi (n = 24 and n = 19, respectively, Fig. 1b, Supplementary Figure S2) and GO term analysis showed that these genes are involved in reactive oxygen species production and cellular catabolic processes, suggesting metabolic deregulation upon viral infection (Supplementary Figure S2). Overall, these results suggest that DCV infection strongly induces the heat shock response in *Drosophila* S2 cells.

### The heat shock response is induced upon DCV and CrPV infection *in vivo*

**Figure 2:**
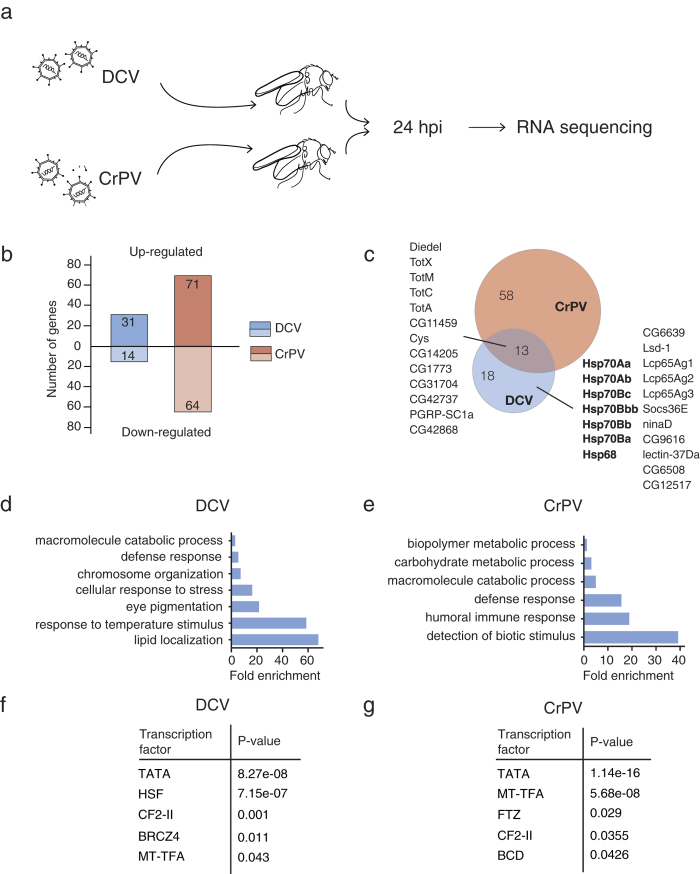
Transcriptome analysis after RNA sequencing of DCV or CrPV-infected flies. (**a**) Overview of the experimental workflow. Female flies (3-5 days old) were inoculated with DCV, CrPV (TCID_50_ = 10,000), or Tris buffer (mock infection) and total RNA was extracted at 24 hpi for next-generation sequencing. Figure drawn by S.H. Merkling. (**b**) Number of differentially expressed genes (≥2-fold over mock) at 24 hpi with DCV or CrPV. (**c**) Venn diagram representing the overlap between the upregulated genes (≥2-fold over mock) upon DCV and CrPV infection. (**d**,**e**) Gene ontology analysis of the genes that are upregulated by (**d**) DCV or (**e**) CrPV at 24 hpi. All significantly enriched level 4 GO terms are shown (*P* < 0.05 in a hypergeometric test with Benjamini & Hochberg correction). (**f**,**g**) Enrichment of predicted transcription factor binding sites amongst genes induced ≥2 fold at 24 hpi with DCV (**f**) or CrPV (**g**). The 500-bp region upstream of the transcriptional start site was analysed in Pscan, using the Transfac database. All transcription factors that are significantly enriched over the genome-wide mean are shown (*P* < 0.05 in a z-test).

To analyze the global transcriptional response to virus infection *in vivo*, we generated genome-wide transcriptomes of DCV-infected wild-type flies at 24 hpi by next-generation sequencing (RNAseq). In addition, we included another member of the *Dicistroviridae* family, Cricket paralysis virus (CrPV) (Fig. 2a). Consistent with our observations in S2 cells (Fig. 1), only few genes were up- (n = 31) or down-regulated (n = 14) after DCV infection (Fig. 2b and Supplementary Figure S3). In contrast, CrPV infection altered the expression of many more genes (n = 71 up, n = 64 down, Fig. 2b and Supplementary Figure S4).

A core set of 13 genes was expressed at >2-fold higher level over mock in both DCV- and CrPV-infected flies (Fig. 2c). These genes included the Jak-Stat dependent and stress-induced genes encoding Diedel and Turandot (Tot) proteins, induction of which has been reported before in DCV infection^8^,^34^,^40^. Another immune gene that was induced by both DCV and CrPV was the peptidoglycan-recognition protein PGRP-SC1a, which is essential for Toll signalling in the context of antibacterial responses^41^. The putative function of the other core genes in host defence has not been evaluated, but they are interesting candidates for follow-up studies. Heat shock proteins were absent from this core set, as they were induced by DCV only (Fig. 2c). Accordingly, GO terms such as “chromosome organization” or “response to temperature stimulus” were significantly enriched among the genes up-regulated by DCV (Fig. 2d), but were absent amongst genes up-regulated by CrPV (Fig. 2e). In line with these observations, analysis of transcription factor binding motifs in DCV-induced genes revealed strong enrichment of the Hsf motif (Fig. 2f), which was absent in the CrPV dataset (Fig. 2g). Other binding motifs, such as Chorion factor 2 and Mitochondrial transcription factor A were enriched in both DCV and CrPV datasets, suggesting that multiple signalling pathways are activated upon viral challenge. The genes that were downregulated upon DCV or CrPV infection were enriched for GO terms such as extracellular matrix and cytoskeleton organization, as well as lipid metabolism (Supplementary Figure S3 and S4). Together, these data suggest that a conserved transcriptional response is induced upon RNA virus infection, complemented with a virus-specific response, as previously suggested^7^. Together, our transcriptome analyses indicate that infection with DCV, but not CrPV induces, the heat shock response *in vivo*.

### Dynamics of the heat shock response upon viral infection in S2 cells

**Figure 3:**
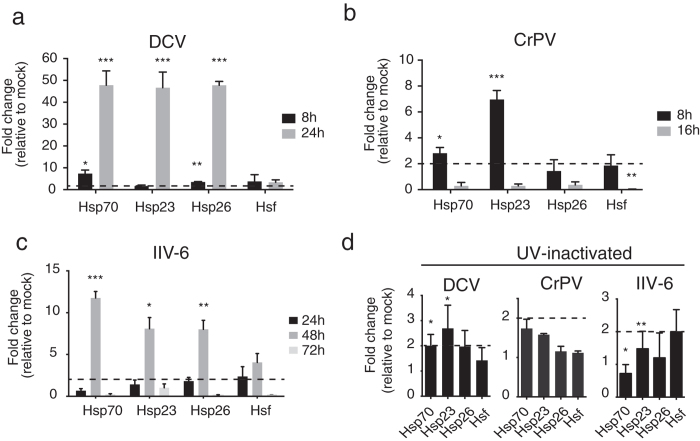
The heat shock response is dynamic and requires viral replication in *Drosophila* S2 cells. Expression of the genes encoding the heat shock proteins Hsp70, Hsp23, Hsp26, and the Heat shock transcription factor (Hsf) was monitored at the indicated time points by RT-qPCR after infection with (**a**) DCV, (**b**) CrPV or (**c**) IIV-6 (MOI = 10). (**d**) S2 cells were inoculated with UV-inactivated viruses and gene expression was measured at 24, 16 or 48 hpi with DCV, CrPV and IIV-6, respectively. Expression of the gene of interest was normalized to the housekeeping gene *Ribosomal Protein 49* and expressed as fold change relative to mock infection. Data are mean and s.d. of three independent infections. Student’s t-tests were used to compare virus-infected samples to mock infections (*P < 0.05, **P < 0.01, ***P < 0.0001). Dotted lines mark the threshold of 2-fold change.

**Figure 4:**
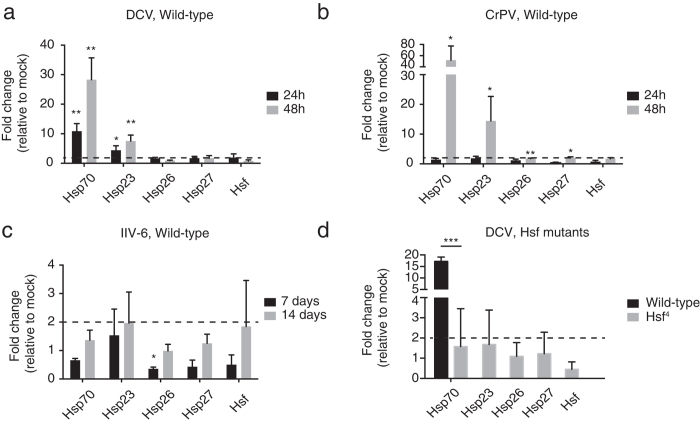
The heat shock response is virus-specific and *Hsf*-dependent *in vivo*. Female wild-type flies (**a**, **b**, **c**) or *Hsf *^4^ mutant flies (**d**) were infected with (**a, d**) DCV, (**b**) CrPV or (**c**) IIV-6. The expression levels of the genes encoding heat shock proteins and Heat shock factor (Hsf) were analysed by RT-qPCR at the indicated time points (**a**, **b**, **c**), and at 24h for DCV in panel **d**. Expression of the gene of interest was normalized to the housekeeping gene *Ribosomal Protein 49* and presented as fold change relative to mock infection. Data are mean and s.d. of three independent experiments. Student’s t-tests were used to compare virus-infected samples to mock infections (*P < 0.05, **P < 0.01, ***P < 0.0001). Dotted lines mark the threshold of 2-fold change.

To validate our results obtained by microarray profiling (Fig. 1) and RNA sequencing (Fig. 2), and to analyze the dynamics of the heat shock response, we performed quantitative RT-PCR (RT-qPCR) on *Drosophila* S2 cells (Fig. 3) or adult flies (Fig. 4) infected with the RNA viruses DCV and CrPV. To test if the heat shock response was specific to RNA virus infection, we included the DNA virus Invertebrate iridescent virus 6 (IIV-6) in our experiments. We first infected S2 cells with DCV and measured expression levels of the genes encoding heat shock transcription factor (*Hsf*) and the heat shock proteins *Hsp70*, *Hsp23*, and *Hsp26* (Fig. 3a). Modest induction of heat shock protein mRNAs was noted at 8 hpi (2 to 7-fold over mock), but very high expression was measured at 24 hpi (45 to 50-fold). In contrast, expression of *Hsf* was slightly elevated (3-fold) at both time points (Fig. 3a).

As we only analysed DCV-infected S2 cells in our microarray analysis, we also tested whether other viruses induce a heat shock response in those cells. After CrPV infection, modest induction of heat shock genes was observed: *Hsp23* was induced 6-fold at 8 hpi, whereas other Hsps and *Hsf* were only induced 2-fold (Fig. 3b). We chose to analyze gene expression at 16 hpi, as cytopathic effect (CPE) is already visible at 24 h after CrPV infection and non-specific RNA degradation may occur at that time point. Strikingly, the heat shock response was no longer induced at 16 hpi with CrPV (Fig. 3b), perhaps due to general inhibition of cellular transcription by CrPV, similar to observations in mammalian picornavirus infections^42^.

Next, we asked whether the DNA virus IIV-6 triggers a heat shock response. As the replication rates of IIV-6 are lower than the ones of DCV or CrPV, we analysed gene expression at 24, 48, and 72 hpi. Expression of the heat shock genes was low at 24 hpi (<2-fold), but was induced at 8 to 11-fold at 48 hpi. However, the response no longer persisted at 72 hpi, which may be due to virus-induced shutdown of host transcription^43^ (Fig. 3c). Similarly, expression of *Hsf* was slightly induced at 24 hpi (2-fold), peaked in expression at 48 hpi (4-fold), before returning to basal levels at 72 hpi (Fig. 3c).

Finally, we tested whether active viral replication was required for heat shock induction. After treatment of S2 cells with UV-inactivated viral particles, the heat shock response remained at basal levels, even at time points with the highest expression upon viral infection (24 h for DCV, 16 h for CrPV and 48 h for IIV-6) (Fig. 3d). These results indicate that viral replication is essential for the induction of the heat shock response.

To confirm that the heat-shock response was not only induced at a transcriptional level, we measured levels of Hsp70 in DCV-infected S2 cells by Western Blotting (Fig. S5), and found induction of Hsp70 at 16 and 24 hpi. Those results were consistent with the data obtained by RT-qPCR and confirmed the induction of Hsp70 proteins upon virus challenge in *Drosophila* S2 cells.

Overall, our results demonstrate that the heat shock response to virus infection is dynamic in magnitude and time. Interestingly, induction of the heat shock response does not correlate with viral RNA loads, as no robust induction was detected at late time points of CrPV and IIV-6 infections, during which higher viral loads are present. This is possibly linked to the global inhibition of host transcription by those viruses^43^,^44^.

### Dynamics of the heat shock response upon viral infection in adult flies

We analysed the dynamics of the heat shock response *in vivo* after systemic infection of adult wild-type flies with DCV, CrPV, or IIV-6. Upon DCV infection, we detected a moderate upregulation of *Hsp23* and *Hsp70* (4 and 10-fold over mock), but not of *Hsp26*, *Hsp27* or *Hsf*. More robust levels of *Hsp23* and *Hsp70* (10 to 30-fold) were measured at 48 hpi, whereas other genes remained at basal levels (Fig. 4a). CrPV infection did not induce the heat shock response at 24 hpi, confirming the outcome of the transcriptome analysis by RNA-seq (Fig. 2). However, strong upregulation of *Hsp23* (14-fold) and *Hsp70* (51-fold) was apparent at 48 hpi (Fig. 4b). Given the lower replication kinetics of IIV-6, we analysed IIV-6 infected flies at 7 dpi when viral loads increased exponentially, and at 14 dpi when titers reached a plateau^33^. However, we detected low levels of heat shock transcripts relative to mock at these time points (Fig. 4c). Thus, although IIV-6 induced transient Hsp expression in S2 cells (Fig. 3c), we were unable to observe a heat shock response in the entire animal. This result suggests that the pathway may not be activated *in vivo*, or that it is induced in specific cell types, such as hemocytes, which would not be detectable by performing RT-qPCR on entire adult flies. Finally, similar to our observations in cells, no significant upregulation of the *Hsf* transcription factor was observed upon DCV, CrPV or IIV-6 infection at the analysed time points.

A previous study found that a subset of heat shock genes could be induced by the JNK pathway and the transcription factor FOXO in response to oxidative stress^45^. We thus asked whether the heat shock response to virus infection was solely dependent on *Hsf* activation. To that end, we exploited the temperature-sensitive *Hsf *^4^ mutant fly, which expresses a mutant Hsf protein with reduced DNA-binding activity, but is viable at 25°C and below^26^. No heat shock genes were induced in *Hsf *^4^ mutant flies upon DCV infection at 24 hpi, whereas the response was very strong in wild-type flies at this time point (Fig. 4d). This is also consistent with previous observations that *Hsp70* induction upon heat stress is *Hsf*-dependent^26^. In conclusion, the heat shock response is induced by RNA virus infection *in vivo* and is dependent on the canonical heat shock transcription factor Hsf.

### The heat shock response is antiviral in *Drosophila*

**Figure 5:**
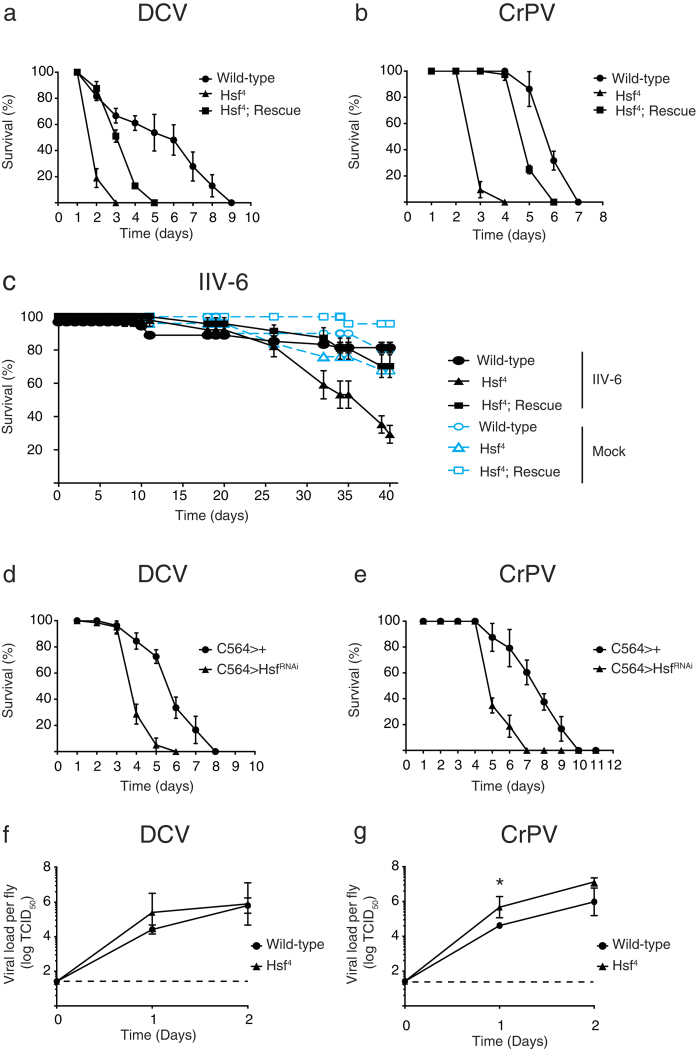
Hsf-deficient flies are hypersensitive to RNA virus infection. (**a-c**) Survival of wild-type flies, *Hsf*^4^ mutants, and *Hsf*^4^ mutants carrying a single *Hsf* transgene (*Hsf*^4^; Rescue) upon (**a**) DCV, (**b**) CrPV, and (**c**) IIV-6 infection. (**d**,**e**) Survival of flies expressing an RNAi-inducing hairpin targeting *Hsf* in the fat body upon (**d**) DCV and (**e**) CrPV infection. The fat body-specific C564 driver line (*C564-Gal4*) was used to drive expression of the transcription factor Gal4, which binds to the Upstream Activating Sequence to induce expression a short hairpin targeting *Hsf* (*UAS-Hsf*^RNAi^). Flies expressing the *C564-Gal4* driver, but not the UAS responder, were included as controls. Mock infections (Tris buffer) were performed along all experiments, and no mortality was noticed over the time course analysed. (**f,g**) Viral titers of wild-type and *Hsf*^4^ mutant flies inoculated with (**f**) DCV or (**g**) CrPV over time. The dashed line represents the detection limit of the titration. Data represent mean and s.d. of three biological replicates of at least 15 female flies for each genotype (**a-e**) or mean and s.d. of three independent experiments, each consisting of 3 replicates of at least 5 female flies for each genotype (**f**,**g**). Differences in viral titers were assessed on log-transformed data with a Student’s t-test (*P < 0.05). Statistical analyses for survival assays (**a**-**e**) are discussed in the main text.

In order to establish whether the heat shock response is important for antiviral defence, or merely a secondary effect of infection-induced stress, we challenged *Hsf *^4^ mutant flies with several viruses and monitored survival over time. We included as genetic control the *Hsf *^4^ mutant carrying a single wild-type *Hsf* transgene under its own regulatory elements (*Hsf *^4^; Rescue). Upon DCV challenge, *Hsf *^4^ mutants succumbed faster to infection than wild-type flies (mean survival = 2.2 and 5.5 days, respectively; *P* < 0.001). The *Hsf *^4^ mutants carrying a rescue construct exhibited a partial rescue of survival (mean survival = 3.5; *P* < 0.001 compared to *Hsf *^4^, Fig. 5a). Similarly, *Hsf *^4^ mutants died prematurely upon CrPV infection (mean survival = 3.1 and 6.2 days for mutant and control flies, respectively; *P* < 0.001). Expression of the *Hsf* transgene rescued the mean survival time close to wild-type levels (5.2 days; *P* < 0.001 when compared to *Hsf *^4^) (Fig. 5b). Finally, we also observed a higher susceptibility of *Hsf *^4^ mutants to IIV-6 infection compared to wild-type flies (*P* < 0.001), whereas the *Hsf* rescue line remained at wild-type levels (Fig. 5c). However, we noted that *Hsf *^4^ mutants also had a reduced life span in mock infections. It is therefore not possible to draw firm conclusions about the role of *Hsf* in IIV-6 infection, as the increased mortality upon IIV-6 infection could be a reflection of the decreased life span of the mutants (Fig. 5c).

To confirm the results obtained with *Hsf *^4^ mutants, we attempted to reduce *Hsf* levels by ubiquitous expression of an RNAi-inducing hairpin RNA (*Hsf  *^RNAi^) using the *Actin*-*Gal4* driver. However, no viable offspring was obtained, consistent with the lethality associated with *Hsf* null alleles^26^. We therefore reduced *Hsf* expression specifically in the fat body using the *C564-Gal4* transgene to drive *Hsf  *^RNAi^ (50% knock-down efficiency, Supplementary Figure S6). These flies showed increased mortality rates compared to control flies both upon infection with DCV (mean survival = 4.2 and 6.0 days; *P* < 0.001) and CrPV (mean survival = 5.5 and 7.8 days; *P* < 0.001) (Fig. 5d,e). In conclusion, impairing the heat shock response using a *Hsf* loss-of-function allele and gene knockdown results in increased sensitivity to viral infection, suggesting that the heat shock response is important for antiviral defence.

Next, we asked whether the increased sensitivity of *Hsf *^4^ mutants to viral infections was accompanied by higher viral replication rates. Moderate differences in viral titers were observed between wild-type and mutant flies during the 2 day time-course following DCV infection, with a 10-fold difference on the first day, which did not reach statistical significance (Fig. 5f). Upon CrPV infection, a small, but significant, increase in viral titers was observed at 1 dpi (10-fold, *P* < 0.05) (Fig. 5f). In conclusion, we demonstrate that the hypersensitivity of *Hsf *^4^ mutants to viral infections is accompanied by a modest increase in viral titers.

### RNAi and inducible immune pathways are functional in *Hsf*-deficient flies

**Figure 6:**
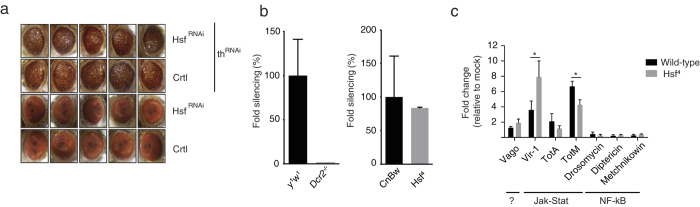
RNAi and inducible immune pathways are functional in *Hsf*-deficient flies. (**a**) Eye phenotype of 5 to 7-day-old flies expressing a hairpin RNA targeting the Drosophila Inhibitor of Apoptosis *thread* (*th*^RNAi^) and *Heat shock factor* (*Hsf  *^RNAi^). The genetic background of the *Hsf  *^RNAi^ line was used as a control (Ctrl). No eye phenotype was observed in control and *Hsf  *^RNAi^ flies that do not expressing the *th*^RNAi^ transgene. Five representative images are shown for each genotype. (**b**) *In vivo* RNAi reporter assay. Reporter plasmids encoding Firefly (Fluc) and Renilla (Ren) luciferase were transfected along with Fluc specific dsRNA or non-specific control dsRNA in *Hsf *^4^ and *Dcr2*^-/-^ mutant flies and their wild-type controls (*CnBw* and *y*^1^*w*^1^, respectively). Reporter gene activity was measured in fly lysates at three days after transfection. Fold silencing by Fluc dsRNA relative to control dsRNA was calculated and presented as the percentage of silencing relative to wild-type controls (see Fig. S6 for Fluc/Ren ratios of all samples). Bars represent mean and s.d. of three pools of five flies for each genotype. (**c**) Expression of inducible immune genes at 24 hpi with DCV (TCID_50_ = 10,000) determined by RT-qPCR in wild-type or *Hsf *^4^ mutant flies. Expression of the gene of interest was normalized to transcript levels of the housekeeping gene *Ribosomal Protein 49* and expressed as fold change relative to mock infection (Tris buffer). Data are mean and s.d. of three independent pools of 15 female flies for each genotype. Student’s t-tests were used to compare the difference in expression between wild-type and mutant flies (*P < 0.05).

RNAi is one of the major antiviral pathways in *Drosophila*^4^,^11^ and its interaction with heat shock components has previously been demonstrated. For instance, the Hsc70/Hsp90 chaperone machinery participates in RISC assembly, and *Hsp70* is transcriptionally regulated by the RNAi machinery^46^,^47^,^48^,^49^. We therefore tested whether the RNAi pathway is fully functional in *Hsf* mutants. We first assayed RNAi activity using an *in vivo* sensor assay^34^ that is based on silencing of the inhibitor of apoptosis *thread* (*th*) by expression of an RNAi-inducing hairpin RNA (*th*^RNAi^)^32^,^50^. Severe apoptosis in the developing eye is triggered by expression of *th*^RNAi^ under control of the eye-specific driver *GMR-Gal4*, resulting in reduced eye size, roughening of the eye surface, and loss of pigmentation in adult flies (Fig. 6a). This phenotype is absent in mutant flies missing the central catalytic component of the pathway, *Argonaute 2* (*AGO2*), indicating that it is fully dependent on the RNAi pathway^32^,^50^. We concomitantly expressed the *th*^RNAi^ and *Hsf  *^RNAi^ hairpins in the eye and monitored the resulting phenotypes. As controls, we included control flies that do not express *Hsf  *^RNAi^, but have the same genetic make-up, as well as flies that do not express *th*^RNAi^. Both in *Hsf  *^RNAi^ and control flies, expression of *th*^RNAi^ resulted in an RNAi-induced eye phenotype (Fig. 6a), which was absent in the control flies devoid of *th*^RNAi^. These results thus suggest that *Hsf  *^RNAi^ mutant flies do not have a defect in RNAi activity.

Additionally, we used a luciferase-based RNAi sensor assay to confirm the efficiency of the RNAi response in *Hsf  *^4^ adult flies^50^,^51^. Three days after *in vivo* transfection with Firefly (Fluc) and *Renilla* luciferase reporter plasmids together with either Fluc-specific dsRNA or control dsRNA, silencing efficiency was measured in fly lysates. *Dicer-2* null mutants and their wild-type controls (*y*^1^*w*^1^) were included to verify that silencing of Fluc expression was RNAi-dependent (Fig. 6b, left panel). In contrast to *Dicer-2* mutants, silencing capacity was intact in *Hsf*^4^ flies (Fig. 6b, right panel), confirming that RNA interference is fully functional in *Hsf*^4^ mutant flies.

Virus infection activates a number of evolutionary conserved immune pathways in *Drosophila*. Upon virus infection, the Jak-Stat pathway mediates induction of *virus induced RNA-1* (*vir-1*), and genes encoding Turandot proteins A and M (*TotA* and *TotM*)^7^,^8^. The NF-κB pathways Toll and Imd regulate expression of genes encoding antimicrobial peptides, such as Drosomycin, Metchnikowin, and Diptericin upon bacterial challenge, and in some cases, upon viral infections^52^,^53^,^54^. *Vago* is induced by DCV infection through an unknown signalling pathway^55^. To assess whether these virus-induced genes were regulated by the heat shock response, we measured their expression levels by RT-qPCR at 24 hours after DCV infection of adult wild-type and *Hsf  *^4^ mutant flies (Fig. 6c). Consistent with previous observations, Jak-stat-dependent genes, especially *vir-1* and *TotM*, were induced by virus infection. *Vir-1* was induced to higher levels in *Hsf  *^4^ mutants than in wild-type flies (7.9-fold and 3.6-fold over mock, respectively, *P* < 0.05), likely caused by higher viral replication levels. *TotM* induction was slightly lower in *Hsf  *^4^ mutants (6 and 4-fold in wild-type and mutant, respectively), whereas *TotA* and *Vago*, as well as antimicrobial peptides were not induced or only to low levels (<2-fold over mock) (Fig. 6c). Overall, no major differences were observed between wild-type and *Hsf  *^4^ mutant flies, indicating that inducible responses to virus infection are not regulated by the heat shock response, and that differences in survival of *Hsf* mutants cannot be explained by defects in known immune pathways.

### Transgenic activation of the heat shock response reduces viral load

Host defence has been proposed to rely on resistance mechanisms that reduce pathogen load and tolerance mechanisms that reduce infection-inflicted damage, but are not directly associated with control of pathogen load^56^,^57^. As *Hsf  *^4^ mutant flies are more sensitive to infection and support mildly higher virus replication than control flies, our data suggest that the heat shock response contributes to resistance to viral infection. To further support this conclusion, we used overexpression studies to deduce whether the heat shock response confers tolerance or resistance to infection. If the heat shock response mediates resistance by driving expression of antiviral effectors, it is expected that transgenic activation of the response would reduce viral replication and prolong survival upon infection.

**Figure 7:**
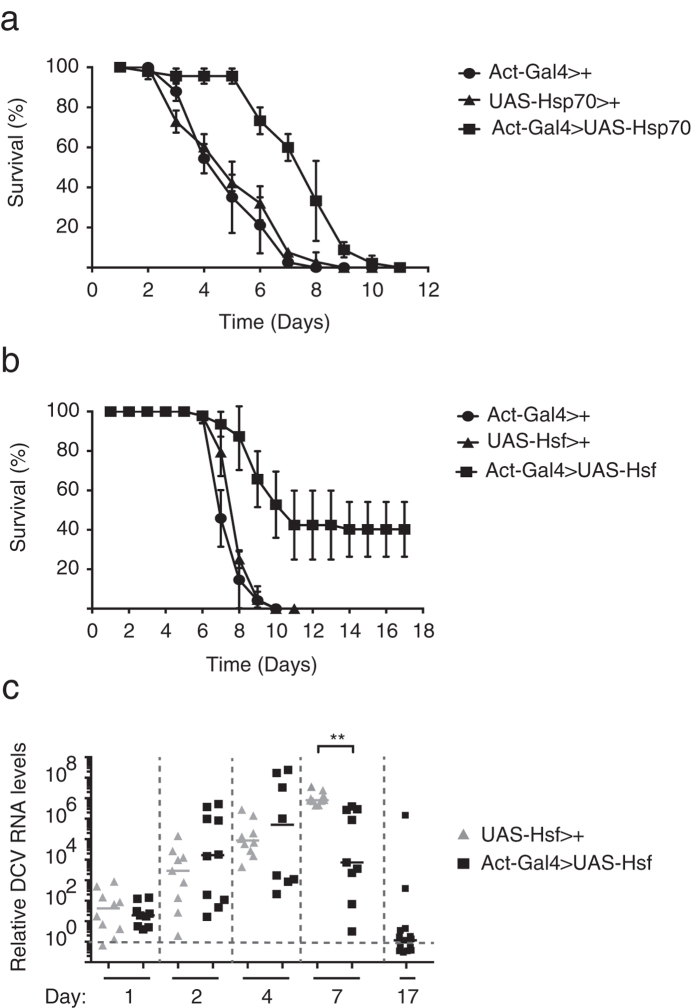
Transgenic activation of the heat shock response protects against viral infection. (**a**,**b**) Survival of flies overexpressing (**a**) *Hsp70* or (**b**) *Hsf*. The ubiquitous driver line (*Actin-Gal4*) was used to drive expression of (**a**) *Hsp70* or (**b**) *Hsf*. Flies expressing only the *Actin-Gal4* driver or the responders *UAS-Hsp70* or *UAS-Hsf* were included as controls. Mock infections (Tris buffer) were performed along all experiments, and no mortality was noticed over the analysed time course. (**c**) Viral RNA load was measured by RT-qPCR in single flies over time. The experiment was performed simultaneously with the survival of panel (**b**). Ten flies were analysed for each time point and genotype. DCV RNA levels were normalized to the housekeeping gene *Ribosomal Protein 49* and presented as fold change over the viral RNA levels in flies harvested immediately after inoculation. A Wilcoxon rank-sum test was used to compare differences in viral load (**P < 0.01). Data represent mean and s.d. of three biological replicates (**a**,**b**) of at least 15 female flies for each genotype.

We tested this hypothesis by ubiquitously overexpressing *Hsf* or *Hsp70* using the *Actin-Gal4* driver. As controls, we included flies expressing either the *Actin-Gal4* driver (*Act-Gal4*>+) or the *UAS-Hsp70* responder (*UAS-Hsp70*>+). Upon DCV infection, we observed lower mortality rates in *Hsp70*-overexpressing flies compared to control flies (mean survival time of 7.6 and 5.1 days, respectively; *P* < 0.001; Fig. 7a). Consistently, mortality rates of flies overexpressing *Hsf* significantly decreased upon DCV infection, compared to *Act-Gal4*>+ controls (mean survival time of 12.5 and 7.6 days, respectively; *P* < 0.001; Fig. 7b).

Interestingly, we observed that a subset of the *Hsf*-overexpressing flies did not succumb to infection. At 17 dpi, about 40% of the flies were still alive and did not exhibit symptoms of pathology, such as abdominal swelling or reduced locomotion. This intriguing observation could indicate that the flies were refractory to the virus, or that virus replication was suppressed to non-pathogenic levels or even fully eradicated. To address this question, we analysed in parallel to the survival assay the viral RNA load by RT-qPCR in single flies at different time points after infection (1, 2, 4 and 7 dpi), and at 17 dpi on the protected flies (Fig. 7c). At 1 dpi, DCV RNA was detectable at low levels, both in control flies and in *Hsf*-overexpressing flies. At 2 and 4 dpi, viral loads increased dramatically in both groups, with *Hsf*-overexpressing flies showing a broad distribution of viral loads, and the control group showing a more homogeneous distribution. At 7 dpi, all *Hsf*-overexpressing flies were still alive and harboured significantly lower viral loads than control flies. In the control group, 55% of the flies already died (Fig. 7b); the remaining flies were moribund and contained very high viral loads (Fig. 7c). Yet, some of the *Hsf*-expressing flies remained heavily infected, and these likely correspond to the population that would succumb to infection after that time. In the group of flies that were protected from infection until day 17, most flies had very low viral loads that were close to, or even below the level of detection (Fig. 7c), indicating that overexpression of *Hsf* leads to long-term suppression of virus replication.

These experiments demonstrate that overexpression of *Hsp70* or *Hsf* confers resistance to viral infection, indicating that the heat shock response has antiviral activity in *Drosophila*. Overexpression of *Hsf* even enables control of viral loads to undetectable levels in a subpopulation of infected flies, raising the exciting possibility that these flies have fully cleared the infection.

## Discussion

Inducible antiviral defence mechanisms remain poorly defined in insects. By combining transcriptome analyses and functional studies *in vivo*, we show in this study that RNA virus infection induces the heat shock response in a dynamic and *Hsf*-dependent manner. *Hsf* loss-of-function and *Hsf* knockdown reduce resistance to viral challenge in adult flies. Conversely, overexpression of *Hsf* induces resistance to infection. Thus, the heat shock response has direct antiviral activity in *Drosophila*.

The mechanism underlying the antiviral activity of the heat shock response remains unknown. The *Hsf* transcription factor may induce expression of direct antiviral effectors. Strikingly, transgenic activation of the heat shock pathway by *Hsf* overexpression induces long-term control of viral replication, in some flies even to undetectable levels, raising the possibility of complete virus eradication. Similarly, overexpression of *Hsp70* also confers resistance to infection, albeit not as strongly as *Hsf* overexpression. These results suggest that Hsp70 itself has antiviral activity, but that Hsf induces additional downstream effectors that mediate antiviral immunity. Alternatively, the heat shock response may function as an alert system that primes the immune system to deploy more rapid and efficient immune responses. For example, it has recently been suggested in mammalian systems that damaged cells release heat shock proteins, which function as damage-associated molecular patterns (DAMPs) that activate immune cells through binding to Toll-like-receptors^58^,^59^,^60^.

The heat shock signalling cascade has been characterized in depth in *Drosophila*, in which it was first discovered with the “puffing” pattern of polytene chromosomes upon heat treatment^61^. The well-described heat shock response, combined with an extensive genetic toolbox, makes *Drosophila* a good model to further study the activation of the heat shock response and putative downstream effector mechanisms in antiviral defence. For example, it would be of interest to analyze whether heat shock proteins are secreted in the hemolymph and whether they signal infection systemically.

Putative target loci bound by Hsf have recently been characterized by genome-wide analyses^62^,^63^,^64^. Epigenetic marks present on the chromosomal landscape influence *Hsf* binding to heat shock element (HSE) target sites. Prior presence of active chromatin marks, such as H3K4 acetylation, are determinants of binding^64^. Chromatin immunoprecipitation combined with genome tiling and cDNA microarrays provided a map of the *in vivo* binding sites of the Hsf in *Drosophila* embryos or Kc cells^62^,^63^. These studies identified as Hsf target sites, chaperone-encoding genes, as well as many genes with other cellular functions, including metabolic and stress-related genes, which are typically activated upon starvation or oxidative stress. These analyses suggest that multiple downstream reponses can be induced by different stressors and that Hsf forms a signalling hub to coordinate these responses.

The molecular mechanisms underlying the activation of the heat shock response upon viral infection remain elusive. Studies in mammalian infection models suggest multiple possibilities. In line with the chaperone function of heat shock proteins, the heat shock response could result from the accumulation of large amounts of unfolded viral proteins in the cytoplasm^22^. In addition, some components of the innate antiviral immune response, such as MDA-5, have aggregation properties that are potent heat shock inducers^65^,^66^. Their accumulation in the cytoplasm upon infection, which is required for their signalling function, could be a trigger for the heat shock response. In addition, it was recently proposed that Dicer-2 and AGO2, two components of the RNAi pathway, regulate expression of transcriptionally active, euchromatic loci, such as *Hsp70*^46^. Intriguingly, many insect viruses including DCV and CrPV encode suppressors of RNAi^4^,^67^,^68^,^69^. Possibly, these proteins could interfere with RNAi-mediated suppression of *Hsp70*, and the heat shock response would therefore act as a counter-counter-defence mechanism that is activated when the pathogen interferes with the primary defence system. A similar concept has recently been discovered in plants^70^.

Translocation of the heat shock transcription factor to the nucleus and binding to chromosomal target loci is an essential step in activation of the heat shock pathway^71^. Surprisingly, only few studies report Hsf translocation upon viral infection, one in adenovirus infection of HeLa cells, and another in vaccinia virus infection of human macrophages^72^,^73^. The translocation of Hsf is difficult to assess, as it can have a nuclear localization at steady state conditions^14^. Live multi-photon imaging of *Drosophila* salivary glands revealed that upon activation, Hsf translocates from the nucleoplasm to chromosomal loci^16^. Hsf translocation can be used as a read-out for the activation of the heat shock response that, although technically challenging, would be worth investigating in virus infection. For instance, it would be of interest to determine whether Hsf translocation could be visualized in adult flies, and whether Hsf translocation occurs only in infected cells, or also in non-infected cells, and whether tissue specific target loci can be defined.

As the heat shock response exerts antiviral activity, it is likely that viruses have evolved counter-defence strategies to impede it. All steps of the heat shock pathway (activation, Hsf translocation, binding to target promoters, as well as expression and function of downstream effectors) are potential targets for interference by viruses. Strikingly, our results indicate that CrPV is a modest inducer of heat shock genes in S2 cells and, *in vivo*, the response seems to be delayed in comparison to DCV. This is consistent with previous observations that CrPV inhibits the heat shock response to elevated temperatures *in vitro* at early time points after infection^44^. It would be of interest to map this activity to individual viral proteins, and to assess the *in vivo* course of infection of virus mutants with defects in this activity. Studying the complex interactions between the heat shock pathway and virus infection will yield fundamental insights into cellular defence mechanisms, and may uncover new targets for the treatment of viral infection.

## Additional Information

**How to cite this article**: Merkling, S. H. *et al*. The heat shock response restricts virus infection in *Drosophila*. *Sci. Rep*. **5**, 12758; doi: 10.1038/srep12758 (2015).

## Supplementary Material

Supplementary Information
