# Supplementary material for: The heat shock response restricts virus infection in Drosophila
Source: Sci Rep. 2015 Aug 3;5:12758. doi: 10.1038/srep12758 (PMC4522674; doi:10.1038/srep12758)
Supplement: Supplementary Information [file srep12758-s1.pdf]

The heat shock response restricts virus infection in *Drosophila*

Sarah H. Merkling[*], Gijs J. Overheul[*], Joël T. van Mierlo[*], Daan Arends[*], Christian Gilissen[†], Ronald P. van Rij[*]

Supplementary Figure legends

Figure S1. List of upregulated genes (> 2-fold over mock) in DCV-infected *Drosophila* S2 cells at (**a**) 8 hours and (**b**) 24 hpi. Probe set ID is the unique identifier given by Affymetrix to the probes on a microarray.

Figure S2. List of downregulated genes (>2-fold over mock) in DCV-infected *Drosophila* S2 cells at (**a**) 8 hours and (**b**) 24 hpi. (**c**) Gene ontology analysis of downregulated genes at 24 hpi. All significantly enriched level 4 GO terms are shown ($P < 0.05$ in a hypergeometric test with Benjamini & Hochberg correction) No significant enrichment was found at 8 hpi.

Figure S3. List of genes that are (**a**) upregulated and (**b**) downregulated (>2-fold over mock) in wild-type flies at 24 hours after DCV infection. (**c**) Gene ontology analysis of downregulated genes. All significantly enriched level 4 GO terms are shown ($P < 0.05$ in a hypergeometric test with Benjamini & Hochberg correction).

Figure S4. List of genes that are (**a**) upregulated and (**b**) downregulated (> 2-fold over mock) in wild-type flies at 24 hours after CrPV infection. (**c**) Gene ontology analysis of downregulated genes. All significantly enriched level 4 GO terms are shown ($P < 0.05$ in a hypergeometric test with Benjamini & Hochberg correction).

Figure S5. Western blot analysis of Hsp70 proteins in DCV infected S2 cells at 16 and 24 hpi. An anti-Hsp70 monoclonal antibody was used to stain the inducible form of Hsp70. The asterisk indicates a nonspecific band and verifies equal loading of the gel. Molecular mass (kDa) is indicated on the left of the blot.

Figure S6. (**a**) *Hsf* expression in flies expressing an RNAi-inducing hairpin targeting *Hsf* (*UAS-Hsf*^{RNAi}) under control of a fat body specific driver (*C564-Gal4*). Data are presented as percentage of *Hsf* expression in control flies expressing the fat body driver but not RNAi-inducing hairpin (*C564-Gal4*>+). A Student's t-test was used to compare the difference in expression (*$P < 0.05$). (**b**) Firefly (FLuc) and Renilla (Ren) luminescence counts and (**c**) FLuc/Ren ratios in flies subjected to *in vivo* transfection with Firefly and Renilla reporter plasmids, together with a dsRNA targeting Firefly luciferase (dsFluc) or a nonspecific dsRNA (dsGFP). Data are means and s.d. of three independent pools of (**a**) 15 or of (**b, c**) 10 female flies for each genotype and condition.

Supplementary Figure S1

a. Up-regulated, 8 hpi

Probeset ID	Flybase ID	Gene name	Fold change
1631628_s_at	FBgn0033520	Peroxiredoxin 2540	32,26
1639571_s_at	FBgn0013276	Heat shock protein 70Ab; Heat shock protein 70Aa	20,84
1626821_s_at	FBgn0013277	Heat shock protein 70Ba	7,15
1632841_x_at	FBgn0013278	Heatshock protein 70Bc; Heat shock protein 70Bb	7,15
1641055_at	FBgn0001224	Heat shock protein 23	7,13
1635044_at	FBgn0001225	Heat shock protein 26	6,63
1635263_at	FBgn0033519	Dmel_CG11825	5,94
1627613_at	FBgn0014865	Metchnikowin	4,39
1634366_at	FBgn0052185	Dmel_CG32185	2,94
1629061_s_at	FBgn0001223	Heat shock protein 22; Heat shock gene 67Bb	2,88
1640881_at	FBgn0035343	Dmel_CG16762	2,58
1628117_at	FBgn0001226	Heat shock protein 27	2,48
1640405_at	FBgn0010497	lethal (2) 01810	2,23
1625265_at	FBgn0035189	Dmel_CG9119	2,19
1638301_at	FBgn0034010	Dmel_CG8157	2,18
1634640_at	FBgn0031693	Probable cytochrome P450 4ac1	2,05

b. Up-regulated, 24 hpi

Probeset ID	Flybase ID	Gene name	Fold change
1639571_s_at	FBgn0013276	Heat shock protein 70Ab; Heat shock protein 70Aa	26,25
1641055_at	FBgn0001224	Heat shock protein 23	15,34
1635044_at	FBgn0001225	Heat shock protein 26	9,91
1629061_s_at	FBgn0001223	Heat shock protein 22; Heat shock gene 67Bb	6,67
635666_at	FBgn0053906	Histone H2B	4,47
1628117_at	FBgn0001226	Heat shock protein 27	3,59
1626821_s_at	FBgn0013277	Heat shock protein 70Ba	3,33
1632023_s_at	FBgn0039911	Dmel_CG1909	2,85
1640231_a_at	FBgn0034493	Dmel_CG8908	2,81
1627129_at	FBgn0000253	Calmodulin	2,69
1629261_at	FBgn0020660	Eukaryotic initiation factor 4B	2,43
1637786_s_at	FBgn0085820	Dmel_CR41610	2,35
1641609_at	FBgn0005630	longitudinals lacking	2,30
1624070_at	FBgn0010408	Ribosomal protein S9	2,18
1641593_at	FBgn0053969	Dmel_CG33969	2,12
1635210_a_at	FBgn0003137	Papilin	2,05
1631797_at	FBgn0029521	Odorant receptor 1a	2,05
1639005_x_at	FBgn0053272	Dmel_CG33272	2,03
1625387_s_at	FBgn0025615	Torsin-like protein	2,02
1627679_at	FBgn0040371	Dmel_CG12470	2,00

Supplementary Figure S2

a. Down-regulated, 8 hpi

Probeset ID	Flybase ID	Gene name	Fold change
1633989_at	FBgn0037290	Dmel_CG1124	3,57
1635227_at	FBgn0001258	Ecdysone-inducible gene L3	3,01
1639737_at	FBgn0085359	Dmel_CG34330	2,90
1635852_at	FBgn0010194	Wnt oncogene analog 5	2,76
1634964_at	FBgn0038565	Dmel_CG7794	2,62
1629479_a_at	FBgn0020303	fledgling of Klp38B	2,56
1636275_a_at	FBgn0260964	Vesicular monoamine transporter	2,54
1624725_at	FBgn0037565	Dmel_CG9626	2,54
1632491_at	FBgn0052985	Dmel_CG32985	2,41
1624591_at	FBgn0031784	Dmel_CG9222	2,40
1635000_at	FBgn0034603	Dmel_CG9480	2,22
1641552_at	FBgn0010019	Cytochrome P450-4g1	2,20
1626442_at	FBgn0031971	Dmel_CG7224	2,19
1628032_a_at	FBgn0051092	Dmel_CG31092	2,19
1626849_at	FBgn0031723	Dmel_CG7251	2,17
1637257_at	FBgn0027601	Dmel_CG9009	2,17
1637632_at	FBgn0031636	Dmel_CG12194	2,13
1623949_s_at	FBgn0052816	Dmel_CG32816	2,09
1639105_at	FBgn0065110	pickpocket 10	2,07
1637544_s_at	FBgn0085436	Dmel_CG34407	2,06
1639537_at	FBgn0028863	Dmel_CG4587	2,02
1625761_a_at	FBgn0035049	Matrix metalloproteinase 1	2,01
1631441_s_at	FBgn0035539	Dmel_CG7447	2,00
1628494_a_at	FBgn0024315	Putative inorganic phosphate cotransporter	2,00

b. Down-regulated, 24 hpi

Probeset ID	Flybase ID	Gene name	Fold change
1626442_at	FBgn0031971	Dmel_CG7224	4,33
1626846_s_at	FBgn0031914	Dmel_CG5973	3,58
1635227_at	FBgn0001258	Ecdysone-inducible gene L3	3,29
1635635_a_at	FBgn0065097	small non-messenger RNA 357	3,27
1631628_s_at	FBgn0033520	Peroxiredoxin 2540	3,09
1632719_at	FBgn0000279	Cecropin C	2,98
1629479_a_at	FBgn0020303	fledgling of Klp38B	2,70
1639737_at	FBgn0085359	Dmel_CG34330	2,58
1623635_at	FBgn0031701	Turandot M	2,29
1630361_at	FBgn0002945	naked cuticle	2,26
1633989_at	FBgn0037290	Dmel_CG1124	2,17
1626892_at	FBgn0086251	deadlock	2,16
1631408_at	FBgn0029123	SoxNeuro	2,14
1624725_at	FBgn0037565	Dmel_CG9626	2,13
1633770_at	FBgn0037646	Dmel_CG11967	2,12
1625840_at	FBgn0037265	Uncharacterized protein CG12001	2,04
1635263_at	FBgn0033519	Dmel_CG11825	2,03
1635852_at	FBgn0010194	Wnt oncogene analog 5	2,02
1641722_at	FBgn0016715	Rhythmically expressed gene 2	2,01

c

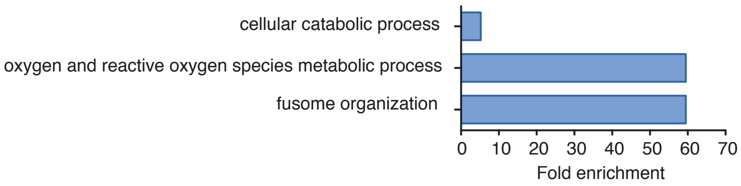

Supplementary Figure S3

a. Up-regulated genes, DCV, 24 hpi

Symbol	Fold change
TotX	32,90
CG11501	20,97
TotM	13,27
Hsp70Aa	9,51
Hsp70Ab	9,45
TotC	7,52
TotA	6,50
Hsp70Bc	6,11
Hsp70Bbb	5,98
Hsp70Bb	5,90
Hsp70Ba	5,86
CG11459	4,76
Hsp68	4,03
CG6639	3,71
CG31704	2,77
Lsd-1	2,68
Lcp65Ag1	2,68
Lcp65Ag2	2,41
CG42868	2,38
Lcp65Ag3	2,36
CG1773	2,22
Socs36E	2,20
ninaD	2,19
Cys	2,13
CG9616	2,13
CG42737	2,13
CG14205	2,10
lectin-37Da	2,10
CG6508	2,06
CG12517	2,03
PGRP-SC1a	2,00

b. Down-regulated genes, DCV, 24 hpi

Symbol	Fold change
Lsp1beta	6,63
Lsp2	4,89
Obp99a	4,06
Cht4	3,73
Fst	3,34
GstD2	2,73
CG16704	2,33
Dro	2,31
Vml	2,28
Vm32E	2,23
CG10621	2,23
CG34291	2,23
ndl	2,08
psd	2,01

c. Gene Ontology
Down-regulated genes, DCV, 24 hpi

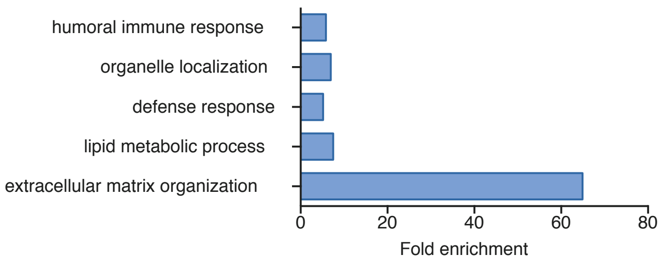

Supplementary Figure S4

a. Up-regulated genes, CrPV, 24 hpi

Symbol	Fold change
CG11501	59,30
TotX	58,08
lectin-24A	38,32
TotM	30,70
Dpt	13,55
AttC	12,91
CG32185	12,82
TotC	12,73
DptB	12,47
TotA	11,96
CG10332/IM18	10,85
CG11459	10,56
CecA1	9,32
AttA	9,25
AttB	9,00
CG33926	5,90
PGRP-SB1	5,86
Mtk	4,79
CG14527	4,41
Cys	4,00
CG14205	3,71
CG14526	3,68
Dro	3,53
Obp56a	3,43
CG1773	3,29
PGRP-SD	3,25
CG3106	3,03
CG5246	2,97
Cyp4d1	2,83
CG31704	2,79
Jon66Cii	2,69
CG4259	2,68
CG42737	2,57
CG9676	2,53
CG9377	2,48
betaTub85D	2,45
CG30431	2,43
LysS	2,43
CG7829	2,43
CG6495	2,39
CG8329	2,39
Npc2d	2,38
Rfabg	2,36
CG13936	2,36
PGRP-SC1a	2,35
CG3239	2,35
CG14225	2,31
CG14872	2,30
PGRP-SC1b	2,22
CG31041	2,20
CG2772	2,17
CG16836	2,16
CG11911	2,16
CG32523	2,14
ap	2,14
Hml	2,13
CG2736	2,13
tim	2,11
CG5618	2,10
CG12057	2,10
CG11912	2,10
GstD5	2,08
Jon66Ci	2,08
Tequila	2,08
CG11852	2,07
CG6361	2,06
CG6295	2,06
CG32368	2,04
CG42868	2,03
sug	2,00
CG17032	2,00

b. Down-regulated genes, CrPV, 24 hpi

Symbol	Fold change
CG7203	14,52
fln	8,28
TpnC4	7,73
Nplp3	6,54
Cht4	6,02
Fst	5,86
Cpr49Ae	4,50
CG9090	4,38
CG14246	3,97
CG3264	3,78
CG15434	3,73
Lsp2	3,71
CG34172	3,58
Mur11Da	3,53
CG4066	3,46
CG15571	3,41
CG14245	3,34
TpnC41C	3,29
CG31926	3,23
CG16772	3,18
CG7409	3,12
Cp19	3,12
CG15065	3,10
CG16704	3,03
CG13084	3,03
Femcoat	2,89
CG13083	2,83
Act79B	2,77
CG12716	2,75
CG9297	2,73
Cp7Fa	2,73
CG2022	2,62
CG4757	2,58
proPO-A1	2,58
Mlc1	2,55
Cp16	2,50
CG11425	2,48
Spn31A	2,46
CG31205	2,45
Mlc2	2,45
Fhos	2,39
CR31084	2,31
Cpr72Ec	2,30
Act88F	2,28
Cpr62Bb	2,28
CG13113	2,27
CG4000	2,25
CG31928	2,20
Npc2e	2,20
CG15528	2,19
Gasp	2,17
Ugt35b	2,16
wupA	2,16
CG34291	2,16
CG16978	2,13
CG34365	2,11
retinin	2,10
CG8193	2,10
Spn43Aa	2,08
CG31904	2,07
Prm	2,04
Pkcdelta	2,04
CG33521	2,03
nAcRbeta-64B	2,00

c. Gene Ontology
Down-regulated genes, CrPV, 24 hpi

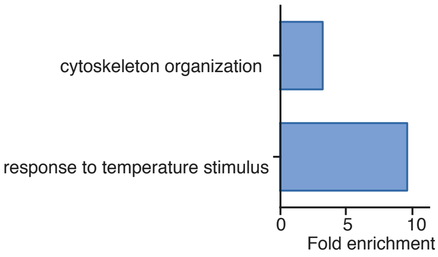

Supplementary Figure S5

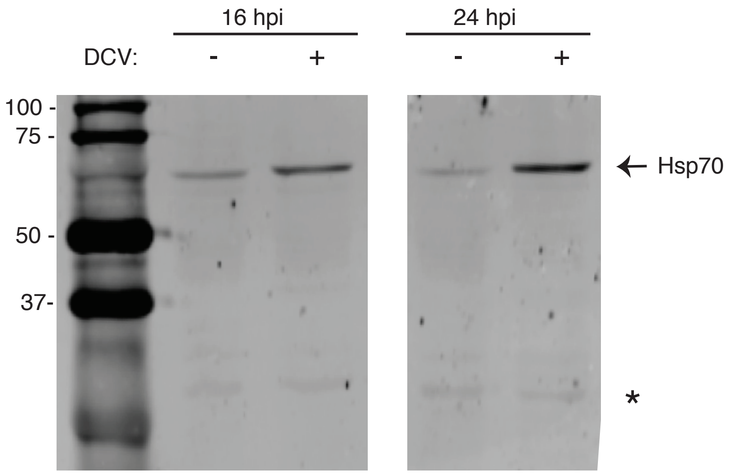

Supplementary Figure S6

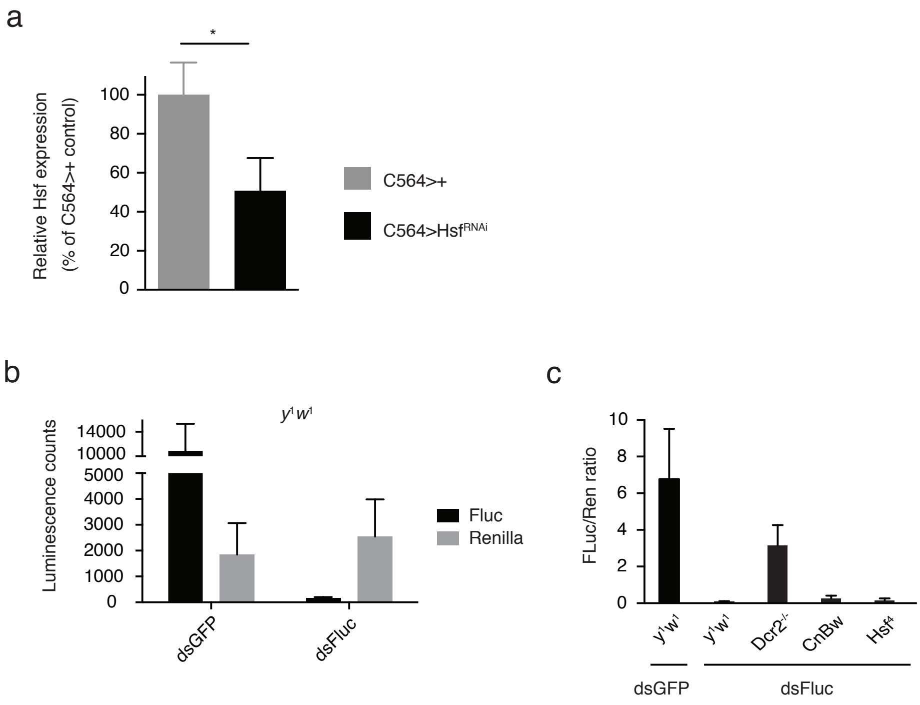

Supplementary Table S1. Primer list.

Target	Primer sequence (5'-3')
Rp49	ATGACCATCCGCCCAGCATAC
	CTGCATGAGCAGGACCTCCA
Hsp70	CACGATGTCGTGGATCTGAC
	GGGCCAAGACTTCTACACCA
Hsp23	TGCCCTTCTATGAGCCCTAC
	TCCTTTCCGATTTTCGACAC
Hsp26	TAGCCATCGGGAACCTTGTA
	GTGGACGACTCCATCTTGGT
Hsp27	GACTGGGTCGTCGTCGTTAT
	ACACCTGGAAGCCATCTTTG
Hsf	GCGATTGACTCACACTTTGG
	TGAGCATTAGCTCGCACAAC
DCV	TTGCCATTGCACCACTAAAA
	AAAATTTCGTTTTAGCCCAGAA
vir-1	ATTACTCCGAATTCGAAGCTTCC
	CGAATTCTTCACGCTCCTTC
TotA	CCCTGAGGAACGGGAGAGTA
	CTTTCCAACGATCCTCGCCT
TotM	ACCGGAACATCGACAGCC
	CCAGAATCCGCCTTGTGC
Vago	CAGCCAAGCGATTCCTTATC
	CTCATACAGTGGGCAGCATC
Drosomycin	GTACTTGTTCGCCCTCTTCG
	ACAGGTCTCGTTGTCCCAGA
Metchnikowin	TACATCAGTGCTGGCAGAGC
	AATAAATTGGACCCGGTCTTG
Diptericin	TGTGAATCTGCAGCCTGAAC
	GCTCAGATCGAATCCTTGCT

Given the high sequence identity amongst members of the Hsp70 genes family, Hsp70 primers detect multiple Hsp70 genes (Hsp70Aa, Hsp70Ab, Hsp70Ba, Hsp70Bb, Hsp70Bc).
